# Supplementary material for: Effectiveness and Safety of Ayurvedic Medicines in Type 2 Diabetes Mellitus Management: A Systematic Review and Meta-Analysis
Source: Front Pharmacol. 2022 Jun 8;13:821810. doi: 10.3389/fphar.2022.821810 (PMC9213670; doi:10.3389/fphar.2022.821810)
Supplement: Supplementary file 1 [file DataSheet1.zip › Supplementary Material/Forest and Funnel Plots/Trigonella foenum-graecum (versus OAD)/LDL-C.pdf]

Trigonella foenum-graecum - LDL-C

	Intervention			Comparator			Mean Diff.	Weight
Study	N	Mean	SD	N	Mean	SD	with 95% CI	(%)
Singh 2016	20	101.03	19.63	20	111.97	28.49	-10.94 [-26.10, 4.22]	95.48
Najdi 2019	5	108.25	31.11	4	154.80	72.55	-46.55 [-116.23, 23.13]	4.52
**Overall**							-12.55 [-27.37, 2.27]	

Heterogeneity: $\tau^2 = 0.00$, $I^2 = 0.00\%$, $H^2 = 1.00$

Test of $\theta_i = \theta_j$: Q(1) = 0.96, p = 0.33

Test of $\theta = 0$: z = -1.66, p = 0.10

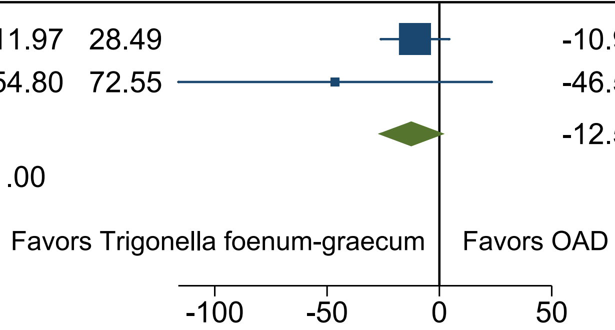

Random-effects REML model
